# Composition optimization of mastic in recycled asphalt mixtures based on pavement performance

**DOI:** 10.1371/journal.pone.0344180

**Published:** 2026-03-06

**Authors:** Xiaohui Li, Zhanghong Liu, Kaimin Fu, Kai Zhang, Maomao Chen

**Affiliations:** 1 Jiangxi Ganyue Expressway Co., LTD, Nanchang, China; 2 Jiangxi Communications Investment Group Co., LTD, Nanchang, China; 3 Jiangxi Provincial Communications Investment Maintenance Technology Group Co., LTD, Nanchang, China; Graphic Era Deemed to be University, INDIA

## Abstract

Due to the depletion of natural sand and gravel resources and increasing environmental restrictions, the utilization of recycled asphalt pavement materials (RAP) has become a critical approach to mitigate the consumption of natural aggregates and reduce carbon emissions in highway construction. The performance of RAP-containing asphalt mixtures is closely associated with the properties of asphalt mastic, which consists of fine RAP (FRAP), fine natural aggregates, mineral filler, and asphalt binder. However, the interactions among these components, namely fine aggregate gradation (calculated via *K* value), mineral filler–binder ratio, and FRAP–fine aggregate ratio, are not yet fully understood, limiting the informed design of asphalt mastic. This study aims to investigate the compositional characteristics of asphalt mastic and propose an optimized gradation suitable for engineering applications. The results indicate that an asphalt mastic with a mineral filler–binder ratio of 1.4, a FRAP–fine aggregate ratio of 50:50, and a *K* value of 0.65 achieves optimal overall mechanical performance. An analysis of variance shows that the mineral filler–binder ratio is the dominant factor affecting mastic performance (p < 0.001), followed by the FRAP–fine aggregate ratio (p < 0.01), while the influence of the *K* value is comparatively weak. Building on these optimized mastic parameters, the effect of the coarse aggregate-to-asphalt mastic ratio was evaluated, with a ratio of 75:25 providing the most balanced mixture performance. Compared with the standard gradation, mixtures designed with the recommended gradation exhibited approximately 35% higher dynamic stability and 28% higher fracture toughness, indicating significantly improved resistance to rutting and cracking.

## Introduction

Due to the need to protect natural resources such as mountain landscapes and vegetation, the mining of natural sand and gravel is restricted, resulting in a shortage of aggregate resources for highway construction. Consequently, the recycling of construction materials without compromising pavement performance has become a key research focus [1–3]. Recycled asphalt pavement materials (RAP), which are obtained through pavement milling, are among the reusable construction materials [4]. In China, the annual production of RAP exceeds 800 million tons [5], and this figure is expected to continue to increase. The reuse of RAP in asphalt mixtures not only reduces the demand for natural aggregates but also decreases carbon emissions, offering significant economic and environmental benefits [6–11].

During the recycling process, RAP is typically classified into two categories based on particle size: coarse RAP (CRAP, with sizes ranging from 19 to 2.36 mm) and fine RAP (FRAP, with sizes ranging from 2.36 to 0.3 mm) [12–16]. Moreover, asphalt mixtures are generally considered three-phase structural systems consisting of a coarse aggregate skeleton, asphalt mastic, and air voids [17,18]. Asphalt mastic, which consists of fine aggregates, mineral filler, and asphalt binder, accounts for approximately 30%–40% of the total mixture mass. CRAP is incorporated into the coarse aggregate skeleton, whereas FRAP is integrated into the asphalt mastic. As a critical component that binds coarse aggregates, the composition and properties of asphalt mastic directly influence the overall mechanical performance and durability of asphalt mixtures [19–22]. The influence of FRAP on the properties of asphalt mastic should be understood, as this knowledge would ensure the pavement performance of asphalt mixtures and enhances the utilization rate of RAP during the design process.

However, the high variability in the asphalt content and aggregate gradation of RAP, along with deviations from the target gradation, has limited its application in high-RAP-content asphalt mixtures [23–25]. Aravindmanikandan et al. [26] found that FRAP can improve the elastic modulus and flow number of asphalt mixtures, indicating potential benefits for high-temperature stability and stiffness. Conversely, Al-Ghurabi et al. [27] argued that FRAP causes insufficient voids in the mineral aggregate, reducing the stability of asphalt mixtures and thereby limiting its practical application. The use of FRAP is constrained by performance-control challenges, underscoring the need for a better understanding of its influence on asphalt mastic properties. Han et al. [28] demonstrated that both low-temperature and fatigue performances decrease, whereas moisture susceptibility initially increase before subsequently declining with increasing proportions of FRAP. Bai et al. [29] concluded that FRAP reduces resistance to low-temperature cracking and repeated loading. Van Winkle et al. [30] found that a high FRAP content improves the high-temperature rutting resistance and increases the stiffness of asphalt mastic, whereas the fracture energy is reduced, indicating a potential increase in susceptibility to low-temperature cracking. Zhang et al. [31,32] indicated that the gradation design parameters exert a significant influence on the sensitivity of FRAP to material performance. These studies showed that FRAP plays an important role in the performance of asphalt mastic. In addition, Rochlani et al. [33], Xing et al. [34], Alzaidy et al. [35], and Al-Mosawe et al. [36] considered that the contents of fine natural aggregates, mineral filler, and asphalt binder also significantly affect the performance of asphalt mastic.

However, existing studies have primarily focused on the influence of FRAP on the performance of asphalt mastic and mixtures while neglecting the analysis of the compositional characteristics of asphalt mastic, specifically, the relationships among FRAP, fine natural aggregates, mineral fillers, and asphalt binders. This limitation restricts the optimal design of asphalt mastic and mixtures that incorporate FRAP.

In response to these limitations, this study investigated the compositional characteristics of FRAP-containing asphalt mastic and their effects on material performance, focusing on factors such as the FRAP–fine aggregate ratio, fine aggregate gradation, mineral filler–binder ratio, and coarse aggregate-to-asphalt mastic ratio. Additionally, a recommended gradation for asphalt mixtures suitable is proposed. These findings provide a foundation for optimizing the design methodology for asphalt mixtures that incorporate FRAP.

## Materials and methods

### Materials

The basic properties of the AH-70 base asphalt were determined according to current Chinese standard “*Standard Test Methods of Bitumen and Bituminous Mixtures for Highway Engineering* (JTG E20-2011)” [37]. The results are presented in Table 1, and all the tested properties satisfied the requirements specified in the Chinese standard “*Technical Specifications for Construction of Highway Asphalt Pavements* (JTG F40-2004)” [38].

**Table 1:** Basic Properties Test Results of Asphalt Binder.

Test property		Test result	Specification
Penetration (25°C, 100g, 5s)/0.1 mm		65	60~ 80
Softening Point (Ring-and-Ball Method)/°C		51.3	≥45
Ductility (5 cm/min, 10°C)/cm		22	≥15
Penetration Index		−0.789	−1.5~+1.0
Dynamic Viscosity at 60°C/(Pa·s)		418	≥180
Density (25°C)/(g/cm^3^)		1.029	N/A
Wax Content		1.53%	<2.2%
After RTFOT	Mass Loss/%	0.34	−1% ~ 1%
	Penetration Ratio/%	62.3%	≥61%
	Ductility (5 cm/min, 10°C)/cm	6.9	≥6

Aggregates in asphalt mixtures are generally categorized into three types: coarse aggregates, fine aggregates, and mineral fillers. The physical and mechanical characteristics of these components significantly affect the performance of asphalt mixtures. Coarse aggregates primarily provide a structural framework, whereas fine aggregates fill the voids within the framework. Mineral fillers, combined with asphalt, form the mastic that occupies the spaces between the coarse and fine aggregates and binds them, thereby enabling the mixture to withstand traffic-induced loads.

Coarse aggregates must exhibit sufficient hardness and strength. Therefore, coarse aggregates with a rough texture, particle shape close to cubic, dry and clean, free from impurities and weathering, and hardness are preferable. The cleanliness of fine aggregates has a significant impact on the adhesion between asphalt and aggregates. Consequently, fine aggregates should be clean, dry, free of soil and impurities, free from weathering, and have appropriate hardness and angular properties. Mineral fillers, as important components of asphalt mixtures, not only serve as fillers but also react with asphalt to enhance its cementing strength. They should be ground from hydrophobic stones such as limestone, be dry and clean, be free from impurities such as soil, and the passing rate of 0.075 mm sieve holes should not be less than 80%.

Limestone aggregates were used in this study. The aggregates are divided into four specifications, namely Aggregate 1 (9.5–19 mm aggregates), Aggregate 2 (4.75–9.5 mm aggregates), Aggregate 3 (stone chips, 2.36–9.5 mm aggregates), and Aggregate 4 (sand, 0.075–2.36 mm aggregates). The screening results for different aggregate specifications are listed in Table 2, and the densities and water absorption rates are listed in Table 3. Limestone powder was used as the mineral filler. The basic properties of the aggregates are listed in Tables 4–6, and all the aggregates were tested according to the current Chinese standard “*Test methods of aggregates for highway engineering* (JTG 3432-2024)” [39].

**Table 2:** Sieve Analysis Results of Aggregates with Different Gradations.

Sieve Opening Size (mm)	Passing Percentage of Aggregates for Different Material Numbers (%)				
	Aggregate 1	Aggregate 2	Aggregate 3	Aggregate 4	Mineral Filler
19	100.0	100.0	100.0	100.0	100.0
16	84.1	100.0	100.0	100.0	100.0
13.2	50.1	99.5	100.0	100.0	100.0
9.5	2.3	82.2	100.0	100.0	100.0
4.75	0.2	0.6	45.2	96.8	100.0
2.36	0.1	0.4	0.4	83.7	100.0
1.18	0.1	0.2	0.2	69.2	100.0
0.6	0.1	0.2	0.1	44.1	100.0
0.3	0.1	0.2	0.1	15.9	100.0
0.15	0.0	0.1	0.0	1.3	100.0
0.075	0.0	0.1	0.0	0.3	88.9

**Table 3:** Density and Water Absorption of Aggregates with Different Gradations.

Aggregate Type	Apparent Density *G*_*a*_(g/cm^3^)	Bulk Density *G*_*b*_(g/cm^3^)	Water Absorption(%)
Aggregate 1	2.633	2.558	0.12
Aggregate 2	2.618	2.564	0.15
Aggregate 3	2.687	2.536	0.30
Aggregate 4	2.492	2.430	0.41
Mineral Filler	2.728	--	--

**Table 4:** Test Results of Coarse Aggregates.

Parameter	Specification	Test Result	Test Method
Aggregate Strength (%)	≤12	7.9	T 0314
Soft Particles Content (%)	≤3	1.5	T 0320
Aggregate Crushing Value (%)	≤26	12.4	T 0316
Los Angeles Abrasion Loss (%)	≤28	14.7	T 0317
Water Absorption (%)	≤2.0	1.3	T0304
Filler Content (<0.075 mm) by Washing (%)	≤1	0.35	T 0310
Flaky and Elongated Particles Content (10–20 mm) (%)	≤12	3.0	T 0312
Flaky and Elongated Particles Content (5–10 mm) (%)	≤18	8.3	T 0312

**Table 5:** Test Results of Fine Aggregate.

Parameter	Specification	Test Result	Test Method
Angularity (Flow Time) (s)	≥30	45	T 0345
Methylene Blue Value (g/kg)	N/A	0.85	T 0346
Sand Equivalent (%)	≥60	76	T 0334
Aggregate Strength (>0.3 mm fraction) (%)	≥12	15.4	T 0340
Fines Content (<0.075 mm fraction) (%)	≤3	1.0	T 0333

**Table 6:** Test Results of Mineral Filler.

Parameter	Specification	Test Result	Test Method
Hydrophilic Coefficient	<1	0.58	T 0353
Moisture Content (%)	≤1	0.38	T 0103
Heating Stability	Measured Observation	Stable	T 0355
Appearance	No Agglomeration	No Agglomeration	N/A
Plasticity Index	<4	3.2	T 0354
Particle Size <0.6 mm (%) Particle Size <0.15 mm (%) Particle Size <0.075mm (%)	100 90 ~ 100 75 ~ 100	100 95.1 87.6	T 0351

The FRAP used in this study was obtained by the milling asphalt surface layers. According to the procedures specified in Chinese standards “*Standard Test Methods of Bitumen and Bituminous Mixtures for Highway Engineering* (JTG E20-2011)” and “*Test Methods of Aggregates for Highway Engineering* (JTG 3432-2024)” [37,39], the reclaimed material was subjected to sieve analysis and its key technical properties were characterized. The results, summarized in Tables 7 and 8, complied with these requirements specified in “*Technical Specifications for Highway Asphalt Pavement Recycling* (JTG/T 5521-2019)” [40].

**Table 7:** Sieve analysis results of FRAP.

Sample ID	Passing percentage under each sieve size (mm) (%)							
	9.5	4.75	2.36	1.18	0.6	0.3	0.15	0.075
1	100.0	89.1	62.8	38.7	17.2	7.2	3.2	0.9
2	100.0	88.6	59.2	36.1	16.3	7.5	3.2	0.8
3	100.0	89.5	64.0	40.5	17.8	7.4	3.1	1.0
4	100.0	88.0	60.8	38.6	17.0	7.3	3.1	0.9
Average	100.0	88.8	61.7	38.5	17.1	7.4	3.2	0.9

**Table 8:** Technical properties of FRAP.

Property	Test result	Specification requirement
Aged asphalt content (%)	6.29	N/A
Moisture content (%)	0.20	≤3
Apparent relative density (g/cm^3^)	2.508	≥2.5
Clay content (%)	3.6	≤3
Sand equivalent (%)	65	≥60

### Experimental methods

The experimental program was designed to address three objectives: (a) optimization of the asphalt mastic composition through an orthogonal experimental design, (b) determination of the optimal coarse aggregate–asphalt mastic ratio, and (c) performance verification of asphalt mixtures.

**Fig 1:**
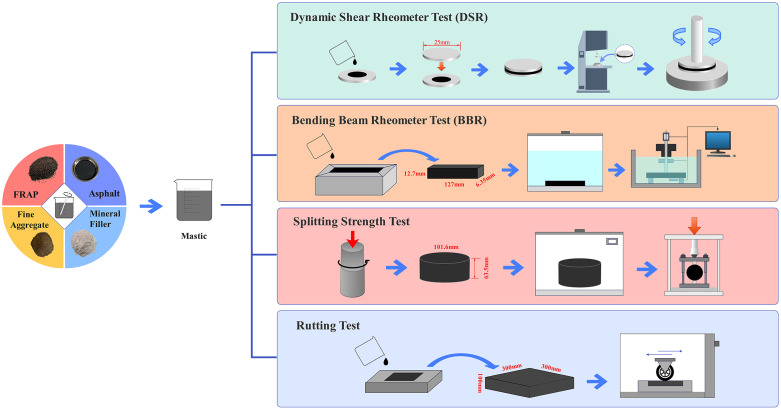
Experimental workflow for asphalt mastic optimization.

For asphalt mastic optimization, the rheological and mechanical properties were evaluated using a Dynamic Shear Rheometer (DSR) test at 58°C, the Bending Beam Rheometer (BBR) test at −18°C, the rutting test at 60°C, and the splitting strength test at −10°C. These tests were employed to characterize the high-temperature, low-temperature, and bonding properties of the mastic. The corresponding experimental workflow is illustrated in Fig 1.

**Fig 2:**
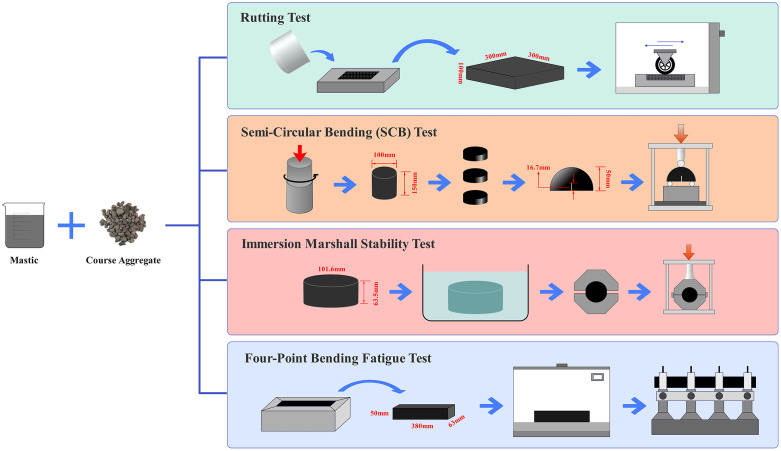
Experimental workflow for asphalt mixture performance evaluation.

To determine the coarse aggregate-to-asphalt mastic ratio and verify the performance verification of asphalt mixtures, we employed the rutting test at 60°C, the semi-circular bending (SCB) test at −10°C, the four-point bending fatigue test at 15°C, and the immersion Marshall stability tests at 60°C to evaluate the rutting resistance, fracture behavior, fatigue life, and moisture susceptibility at the mixture level. The corresponding experimental workflow is illustrated in Fig 2.

For the preparation of all asphalt mixture specimens, the mixing and compaction temperatures were controlled within the ranges of 140–160°C and 120–150°C, respectively. All specimen preparation procedures, test conditions, and data acquisition methods strictly followed the relevant Chinese standard “*Standard test methods of bitumen and bituminous mixtures for highway engineering* (JTG E20-2011)” [37], ensuring consistency and reproducibility.

## Result and discussion

### Composition analysis of asphalt mastic

The performance of asphalt mastic composed of asphalt binder, mineral filler, and fine aggregates (0.075–2.36 mm) was investigated through laboratory tests.

To ensure a rational experimental design, we first determined the factors considered in the orthogonal test based on the compositional mechanism of asphalt mastic. The asphalt mastic studied was a composite system consisting of an asphalt binder, a mineral filler, and fine aggregates (including FRAP and natural fine aggregates). Therefore, pavement performance is governed primarily by the relative proportions of these constituents rather than by the dosage of a single material.

Accordingly, three factors with clear physical meaning and engineering relevance were selected for the orthogonal design: (a) mineral filler–binder ratio, which controls the mastic stiffening and the balance between rutting resistance and brittleness; (b) FRAP–fine aggregate ratio, which represents the replacement degree of natural fine aggregates and reflects the contribution of the aged binder coating and aggregate surface characteristics; and (c) *K* value, used to calculate the fine aggregate gradation, which characterizes the particle size distribution and affects the packing structure and bonding behavior. The dominance and suitable ranges of these factors were preliminarily screened using single-factor comparative tests (Tables 9–12), based on which a subsequent orthogonal experimental design was developed.

#### Determination of the test level.

(1) Mineral filler–binder ratio

The binder content of asphalt mastic directly determines the effective thickness of the asphalt film. An appropriate film thickness enhances the strength and stability of the material, whereas an excessive film content results in surplus asphalt and reduced performance, and an insufficient film content provides inadequate bonding, thereby diminishing the properties of the material. Table 9 presents the test results of the asphalt mastic at different mineral filler-to-asphalt ratios, which were used to determine the test levels for this parameter. Here, *C*_*V*_ represents the coefficient of variation, and its calculation is given by Eq. (1):

**Table 9:** Performance of asphalt mastic under different mineral filler–binder.

Mineral filler–binder ratio	DSR		BBR	
	*A*_*V*_ (kPa)	*C*_*V*_ (%)	*A*_*V*_ (MPa)	*C*_*V*_ (%)
0.6	2.51	3.74	125	6.21
0.8	2.60	8.41	140	5.74
1.0	3.00	5.63	160	7.80
1.2	3.50	2.56	280	4.74
1.4	3.90	7.25	420	3.42
1.6	4.10	5.12	465	4.83
1.8	4.25	6.48	500	2.65
2.0	4.32	5.69	505	5.16


$$C_{V}=\frac{s}{A_{V}}\times 100\%$$ (1)

where, *C*_*V*_ represents the coefficient of variation, *s* is the sample standard deviation, and *A*_*V*_ is the average value of the test results.

As shown in Table 9, when the mineral filler**–**binder ratio was less than 1.0, the complex shear modulus obtained from the DSR tests at 58°C remained relatively low, indicating that the stiffening effect of the mineral filler was insufficient to provide adequate high-temperature resistance. As the ratio increased from 1.0 to 1.4, the complex shear modulus exhibited a pronounced increase, reflecting enhanced high-temperature stability owing to improved mineral filler**–**binder interactions. However, beyond a ratio of 1.4, the growth rate of the complex shear modulus decreased markedly, suggesting gradual saturation of the mineral filler stiffening effect. In contrast, the creep stiffness obtained from the BBR tests at −18°C showed increased sensitivity at higher mineral filler**–**binder ratios. When the ratio exceeded approximately 1.2, the creep stiffness increased more rapidly, reflecting an increased low-temperature stiffness and a potential risk of brittleness.

Considering the different sensitivity characteristics of high- and low-temperature performances, four representative mineral filler–binder ratios (1.0, 1.2, 1.4, and 1.6) were selected. These levels respectively represent a baseline condition, transition point for low-temperature stiffness, near-optimal high-temperature performance state, and upper-bound condition approaching mineral filler saturation, ensuring that the orthogonal experiment captures the most performance-sensitive region without extending into ineffective extremes.

(2) Fine aggregate gradation

Fine aggregate gradation plays a critical role in governing the internal structure and load-transfer characteristics of asphalt mastic, and its rational design is essential for enhancing its mechanical performance. Accordingly, in accordance with the Chinese standard “*Technical specifications for construction of highway asphalt pavements* (JTG F40-2004)” [38], the *K* value was introduced as a design parameter for fine aggregate gradation. Based on a given *K* value, the corresponding fine aggregate gradation was uniquely determined using Eqs. (2)-(4). In other words, each specified *K* value corresponded to one unique fine aggregate gradation. In the orthogonal experiments, different *K* values were prescribed to generate the corresponding fine aggregate gradation schemes, which were then subjected to laboratory tests to systematically investigate the relationship between the given *K* values and asphalt mastic performance, with the aim of identifying an appropriate gradation range.

The gradation of the fine aggregates was determined using the *K* method, whereas the passing rates of aggregates of various sizes were calculated using Eq. (2).


$$P_{x}\text{=}(1-\frac{1-K^{x}}{1-K^{n}})\times 100\%$$ (2)

where, *P*_*x*_ represents the passing rate of the specification aggregates. *K* represents the coefficient of mass decrement. The coefficients *x* and *n* are calculated using Eqs. (3) and (4), respectively.


$$x\text{=3.32}\times \text{lg}\frac{D_{\text{1}}}{d_{x}}$$ (3)

$$n\text{=3.32}\times \text{lg}\frac{D_{\text{1}}}{D_{n}}$$ (4)

where, *D*_1_ is the maximum particle size, *D*_*n*_ is the minimum particle size, and *d*_*x*_ is the diameter of the sieve hole.

Based on the above gradation calculation method, the fine aggregate gradations corresponding to different *K* values are summarized in Table 10. As the *K* value increased from 0.50 to 0.85, the overall gradation gradually shifted from coarse to fine, accompanied by a pronounced increase in the proportion of fine particles. For example, when *K* = 0.50, the content passing through the 0.075 mm sieve was only 6.8%, indicating a relatively coarse gradation; whereas when *K* = 0.85, the content passing through the 0.075 mm sieve increased to 19.1%, corresponding to a much finer gradation. These differences in gradation provided a basis for evaluating the effect of fine aggregate gradation on the mechanical performance of asphalt mastic, which was examined through subsequent laboratory testing.

**Table 10:** Fine aggregate gradation under different *K* values.

*K* value	1.18 ~ 2.36 mm	0.6 ~ 1.18 mm	0.3 ~ 0.6 mm	0.15 ~ 0.3 mm
0.50	53.4	26.2	13.7	6.8
0.55	49.6	26.8	15.2	8.4
0.60	46.0	27.1	16.8	10.1
0.65	42.7	27.2	18.2	11.9
0.70	39.6	27.1	19.6	13.7
0.75	36.7	26.9	20.8	15.6
0.80	34.0	26.6	21.9	17.5
0.85	31.6	26.2	23.1	19.1

Based on the preliminary screening results, the mineral filler–binder ratio and the FRAP –fine aggregate ratio were fixed at 1.4 and 50:50, respectively, to isolate the influence of fine aggregate gradation. Subsequently, Marshall specimens of asphalt mastic were prepared and tested for their splitting strength. Table 11 presents the results.

**Table 11:** Test results of splitting strength at different *K* values.

*K* value	Splitting strength	
	*A*_*V*_ (MPa)	*C*_*V*_ (%)
0.50	0.65	3.18
0.55	0.68	2.52
0.60	0.75	2.27
0.65	0.85	4.71
0.70	0.82	3.58
0.75	0.80	4.49
0.80	0.76	6.83
0.85	0.72	4.22

To determine the appropriate *K* value, we conducted preliminary screening tests by varying *K* over a wide range. The results indicated that relatively low *K* values produce an excessively coarse gradation with an insufficient proportion of fine particles, which may reduce the packing density and weaken interparticle bonding. Conversely, relatively high *K* values result in overly fine gradation, increasing binder demand, and potentially compromising structural stability. Within an intermediate range of *K* values, the mechanical performance of the asphalt mastic exhibited a clear sensitivity to gradation variation. In particular, the splitting strength reached a maximum at approximately *K* = 0.65, whereas deviations toward either lower or higher *K* values led to a gradual reduction in performance. This behavior suggests the existence of an optimal gradation region, in which the particle packing and bonding conditions are well balanced. Hence, four representative *K* levels (0.60, 0.65, 0.70, and 0.75) were selected for orthogonal experiments. These levels effectively capture the influence of fine aggregate gradation on the asphalt mastic performance while avoiding excessively coarse or fine gradations.

(3) FRAP–fine aggregate ratio

The FRAP–fine aggregate ratio represents the degree of replacement of natural fine aggregates by fine RAP aggregates and reflects the combined effects of the aged binder coating and aggregate surface characteristics on the asphalt mastic performance. To evaluate the effect of the FRAP–fine aggregate ratio independently, we fixed the mineral filler–binder ratio and *K* value at 1.4 and 0.65, respectively. The proportions of FRAP–fine aggregates and natural fine aggregates within the asphalt mastic were varied to evaluate the dynamic stability and splitting strength; the results are presented in Table 12.

**Table 12:** Test results of asphalt mastic with different FRAP–fine aggregate ratios.

FRAP–fine aggregate ratio	Dynamic stability		Splitting strength	
	*A*_*V*_ (times)	*C*_*V*_ (%)	*A*_*V*_ (MPa)	*C*_*V*_ (%)
80:20	690	6.25	0.57	5.83
70:30	720	5.13	0.60	3.67
60:40	880	7.32	0.68	1.54
50:50	1480	6.55	0.92	2.89
40:60	1020	4.90	0.70	2.36
30:70	600	8.62	0.45	5.93
20:80	580	3.47	0.41	4.35

The results indicate that the asphalt mastic performance exhibits a non-monotonic response to FRAP content. At relatively low FRAP contents, the contribution of the aged binder is limited, whereas at higher FRAP contents, excessive aged binder and increased surface heterogeneity may adversely affect both high- and low-temperature performances. As shown in Table 12, both the dynamic stability and splitting strength reached favorable or near-optimal values when the FRAP–fine aggregate ratio approached 50:50. Deviations toward either a lower or higher FRAP content led to a gradual reduction in performance, indicating the existence of a balanced FRAP content range in which the beneficial stiffening effect of the aged binder and the structural contribution of natural fine aggregates were well coordinated. Hence, four representative FRAP–fine aggregate ratios (30:70, 40:60, 50:50, and 60:40) were selected for the orthogonal experiments. These levels symmetrically covered the performance-sensitive region around the optimal ratio and enabled a systematic evaluation of the FRAP replacement effects without extending into extreme conditions.

#### Orthogonal experiment and analysis.

A three-factor four-level orthogonal experimental design was used. The experimental factors and levels are listed in Table 13. The design scheme is outlined in Table 14, and the test results are presented in Table 15.

**Table 13:** Orthogonal test levels and factors.

Test grade	Test factors and levels		
	*K* value	FRAP–fine aggregate ratio	Mineral filler–binder ratio
Level I	0.60	60:40	1.0
Level II	0.65	50:50	1.2
Level III	0.70	40:60	1.4
Level IV	0.75	30:70	1.6

**Table 14:** Orthogonal experimental design.

Test Number	*K* value	FRAP–fine aggregate ratio	Mineral filler–binder ratio	Orthogonal composition
1	0.60	60:40	1.0	A_1_B_1_C_1_
2	0.60	50:50	1.2	A_1_B_2_C_2_
3	0.60	40:60	1.4	A_1_B_3_C_3_
4	0.60	30:70	1.6	A_1_B_4_C_4_
5	0.65	60:40	1.6	A_2_B_1_C_4_
6	0.65	50:50	1.4	A_2_B_2_C_3_
7	0.65	40:60	1.2	A_2_B_3_C_2_
8	0.65	30:70	1.0	A_2_B_4_C_1_
9	0.70	60:40	1.2	A_3_B_1_C_2_
10	0.70	50:50	1.0	A_3_B_2_C_1_
11	0.70	40:60	1.6	A_3_B_3_C_4_
12	0.70	30:70	1.4	A_3_B_4_C_3_
13	0.75	60:40	1.4	A_4_B_1_C_3_
14	0.75	50:50	1.6	A_4_B_2_C_4_
15	0.75	40:60	1.0	A_4_B_3_C_1_
16	0.75	30:70	1.2	A_4_B_4_C_2_

**Table 15:** Orthogonal test results.

Test Number	Dynamic stability		Splitting strength	
	*A*_*V*_ (times)	*C*_*V*_ (%)	*A*_*V*_ (MPa)	*C*_*V*_ (%)
1	873	3.62	0.97	6.28
2	1226	7.27	1.17	9.12
3	1376	2.15	1.30	2.57
4	859	5.83	0.81	5.48
5	791	1.44	0.91	7.93
6	1489	8.90	1.45	3.27
7	1159	4.36	1.12	1.99
8	960	6.71	0.85	8.53
9	1139	9.38	0.94	4.18
10	1109	2.81	1.08	6.91
11	934	5.17	0.96	6.08
12	1267	7.53	1.16	2.33
13	1321	3.98	1.05	5.60
14	1042	1.74	1.01	7.37
15	986	8.33	0.90	7.02
16	1068	4.76	0.95	3.37

**Fig 3:**
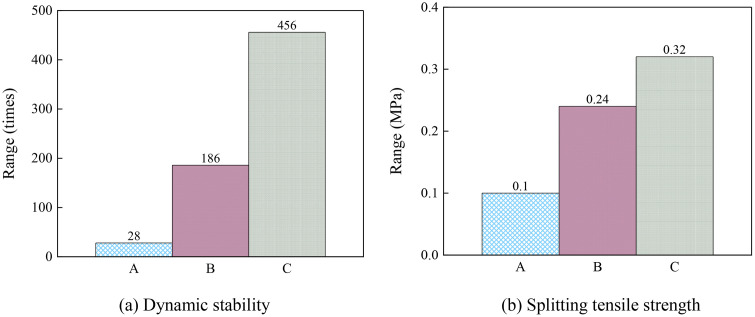
Range.

**Fig 4:**
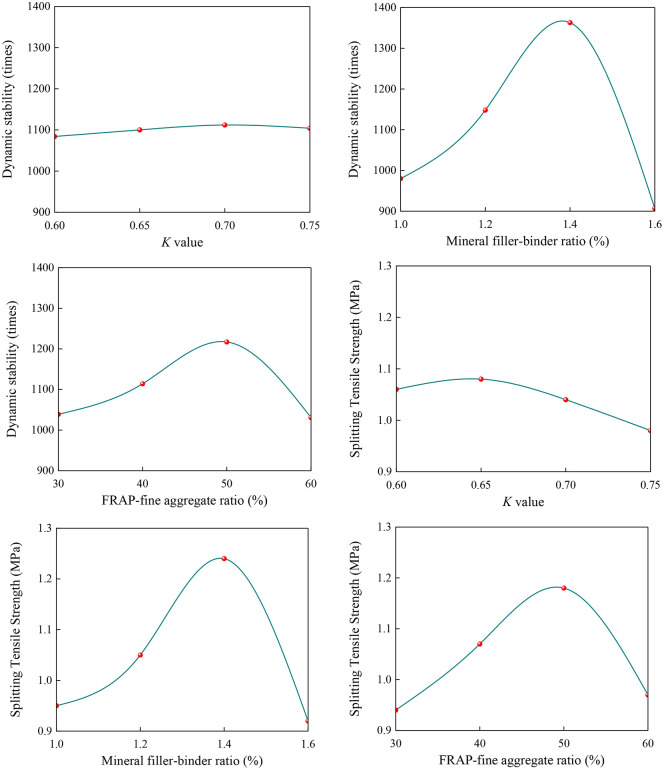
Trend of Change.

In the orthogonal experiments, the mean value of the test results and corresponding range were the two primary indicators listed in Table 16. This range was used to evaluate the degree of influence of different test factors on the results, as shown in Fig 3. This was defined as the difference between the mean values of the different test levels under the same factor. The mean value illustrated the variation trend of each factor across the different levels, as shown in Fig 4.

**Table 16:** Range analysis results.

Performance indicators			*K* value	FRAP–fine aggregate ratio	Mineral filler–binder ratio
Dynamic stability (times)	Average value	Test level I	1084	1031	980
		Test level II	1100	1217	1148
		Test level III	1112	1114	1363
		Test level IV	1104	1039	907
	Range		28	186	456
Splitting strength (MPa)	Average value	Test level I	1.06	0.97	0.95
		Test level II	1.08	1.18	1.05
		Test level III	1.04	1.07	1.24
		Test level IV	0.98	0.94	0.92
	Range		0.10	0.24	0.32

The performance of the mastic changed most significantly with different mineral filler–binder ratios, while the changes were least obvious under different fine aggregate gradations. The fine aggregate gradation exhibited a relatively weaker effect on the high-temperature performance of the mastic. As shown in Fig 4, both the dynamic stability and splitting strength of the asphalt mastic reached their maximum values at a mineral filler–binder ratio of 1.4, indicating that this ratio was critical for achieving optimal performance. Moreover, the asphalt mastic exhibited the best overall behavior when the proportion of asphalt mastic to fine aggregate was 50:50. Regarding the *K* value, the dynamic stability was highest at 0.70, whereas the splitting strength peaked at 0.65. Range analysis further demonstrated that the *K* value had a minimal impact on the dynamic stability; thus, a value of 0.65 was adopted.

Based on the range analysis, which identified the influence trends and optimal levels of the considered factors, an analysis of variance (ANOVA) was conducted to quantitatively evaluate the statistical significance of each factor on the performance indices. The ANOVA results are summarized in Table 17.

**Table 17:** ANOVA results of orthogonal experiment.

Performance indicators		SS	df	MS	F	P-value
Dynamic stability (times)	*K* value	1762	3	587	0.30	0.828
	FRAP–fine aggregate ratio	89218	3	29739	14.93	0.003
	Mineral filler–binder ratio	491884	3	163961	82.30	<0.001
Splitting strength (MPa)	*K* value	0.03	3	0.01	2.51	0.156
	FRAP–fine aggregate ratio	0.14	3	0.05	13.87	0.004
	Mineral filler–binder ratio	0.25	3	0.08	24.85	<0.001

The results indicate that the mineral filler–binder ratio had an extremely significant effect on both the dynamic stability and splitting tensile strength (p < 0.001), confirming that it is the dominant factor governing asphalt mastic performance. The FRAP–fine aggregate ratio also had a statistically significant influence on both performance indices (p < 0.01). In contrast, the *K* value did not pass the significance test, indicating a weaker effect. These findings were consistent with the factor influence ranking obtained from the range analysis, and they further validated, from a statistical perspective, the critical roles of the mineral filler–binder ratio and FRAP–fine aggregate ratio in performance optimization.

In summary, the range analysis and ANOVA results of the orthogonal experiment indicated that the mineral filler–binder ratio exerted the most significant influence on the asphalt mastic performance, followed by the FRAP–fine aggregate ratio, whereas the effect of the *K* value was comparatively weak. The optimal combination was identified as a mineral filler–binder ratio of 1.4, FRAP–fine aggregate ratio of 50:50, and *K* value of 0.65. Under this optimal combination, the asphalt mastic exhibited the highest dynamic stability and splitting tensile strength.

#### Independent verification of optimal combination.

A new batch of asphalt mastic using FRAP collected from different sections of the same highway is prepared to validate the optimal combination. Seven mixtures with varying factor levels were designed as listed in Table 18. The measured dynamic stabilities and splitting tensile strengths are listed in Table 19.

**Table 18:** Test levels of the verification experiment.

Test grade	Test factors and levels		
	Mineral filler–binder ratio	FRAP–fine aggregate ratio	*K* value
Ⅰ (Optimal Composition)	1.40	50:50	0.65
Ⅱ	1.30	50:50	0.65
Ⅲ	1.50	50:50	0.65
Ⅳ	1.40	45:55	0.65
Ⅴ	1.40	55:45	0.65
Ⅵ	1.40	50:50	0.68
Ⅶ	1.40	50:50	0.62

**Table 19:** Verification test results of the optimal combination.

Test Number	Dynamic stability		Splitting strength	
	*A*_*V*_ (times)	*C*_*V*_ (%)	*A*_*V*_ (MPa)	*C*_*V*_ (%)
Ⅰ	1464	3.21	1.42	4.33
Ⅱ	1279	9.67	1.25	1.19
Ⅲ	1252	7.93	1.23	8.55
Ⅳ	1405	5.48	1.37	6.74
Ⅴ	1386	1.82	1.35	2.35
Ⅵ	1453	2.07	1.39	7.12
Ⅶ	1423	6.54	1.41	5.48

As shown in Table 19, after performance testing of asphalt mastic prepared using different batches of raw materials, the mastic produced with the recommended formulation still exhibited the best overall performance among all tested mastics. These results further demonstrated the reliability of the proposed optimized formulation. In addition, the observed effects of the investigated factors (i.e., mineral filler–binder ratio, fine aggregate gradation, and FRAP–fine aggregate ratio) on asphalt mastic performance were likely associated with changes in asphalt film characteristics, particle packing conditions, and interfacial bonding behaviour within the mastic. These aspects will be systematically analyzed in subsequent studies.

### Effect of the coarse aggregate-to-asphalt mastic ratio

The proportions of coarse aggregate-to-asphalt mastic were 90:10, 85:15, 80:20, 75:25, 70:30, 65:35, and 60:40. The gradation of the asphalt mastic was based on the analysis presented in Section 3.1. The recommended gradations are presented in Table 20.

**Table 20:** Proposed gradations for different coarse aggregate-to-asphalt mastic ratios.

Coarse aggregate -to-asphalt mastic	Pass rate (%)									
	19mm	13.2mm	9.5mm	4.75mm	2.36mm	1.18mm	0.6mm	0.3mm	0.15mm	0.075mm
90:10	100.0	63.0	38.0	17.0	10.5	7.5	5.5	4.3	3.6	3.2
85:15	100.0	65.5	41.8	22.0	15.5	11.2	8.2	6.5	5.4	4.8
80:20	100.0	67.7	45.3	26.8	20.5	14.9	11.0	8.7	7.3	6.6
75:25	100.0	69.8	48.8	31.5	25.5	18.5	13.8	10.9	9.1	8.3
70:30	100.0	71.8	52.3	35.9	30.2	22.1	16.5	13.2	11.0	9.9
65:35	100.0	73.9	55.9	41.0	35.2	25.8	19.4	15.4	12.8	11.5
60:40	100.0	76.0	59.3	45.2	40.3	29.4	22.2	17.6	14.6	13.1

The performance indicators of asphalt mixtures with varying ratios of coarse aggregate-to-asphalt mastic are listed in Table 21.

**Table 21:** Performance of asphalt mixtures at different coarse aggregate-to-asphalt mastic ratios.

Coarse aggregate-to-asphalt mastic	Dynamic stability		Fracture toughness		Fatigue life		Immersion Marshall stability	
	*A*_*V*_ (times)	*C*_*V*_ (%)	*A*_*V*_ (MP·m^0.5^)	*C*_*V*_ (%)	*A*_*V*_ (cycles)	*C*_*V*_ (%)	*A*_*V*_ (%)	*C*_*V*_ (%)
90:10	1922	2.47	0.99	3.39	79864	5.91	75.8	6.28
85:15	2738	3.08	1.20	1.92	123574	6.72	86.1	5.19
80:20	3131	5.93	1.32	4.74	195006	7.03	91.5	4.84
75:25	3410	1.64	1.48	1.31	210250	5.47	93.1	6.29
70:30	2770	4.56	1.14	2.36	177318	6.28	91.2	7.89
65:35	2110	2.18	0.92	4.02	142424	5.66	88.9	1.58
60:40	1653	3.95	0.83	3.44	111210	6.95	87.1	8.09

As shown in Table 21, the asphalt mixture exhibited superior pavement performance at a coarse aggregate-to-asphalt mastic ratio of 75:25. Therefore, 75:25 was selected as the optimal coarse aggregate-to-asphalt mastic ratio.

To establish a quantitative linkage between asphalt mastic composition and mixture-level mechanical performance (see Table 21), regression analysis was performed to correlate asphalt mastic content with key performance indicators of asphalt mixtures, as shown in Table 22. The asphalt mastic content was expressed as a percentage of the total mixture mass (*x*, %). *DS*, *K*_*IC*_, *N*_*f*_, and *RMS* represented dynamic stability, low-temperature fracture toughness, fatigue life, and immersion Marshall stability, respectively. The resulting regression equations along with the corresponding coefficients of determination (R^2^) are presented in Table 22. The R² values exceed 0.82, indicating a significant correlation between mixture performance and mastic content.

**Table 22:** Relationship between mixture performance and mastic content.

Relationship between mixture performance and mastic content	R^2^
$DS=-6.4x^{\text{2}}+303.4x-400.7$	0.9285
$K_{IC}=-0.002x^{\text{2}}+0.09x+0.3$	0.8297
$N_{f}=-477.4x^{\text{2}}+24686.1x-122495.7$	0.9164
$RMS=-0.05x^{\text{2}}+2.8x+54.2$	0.9347

### Experimental verification of asphalt mixture gradation

The gradation ranges derived from the test results are listed in Table 23. Compared with the standard gradation, a “two more, one less” strategy is proposed. Specifically, the coarse aggregate content was increased, reflecting its critical contribution to the overall mixture performance; the mineral filler content was also elevated, as the mineral filler–binder ratio plays a decisive role in the properties of the mastic, whereas the fine aggregate content was reduced, given its limited effect on both the aggregate structure and mastic behavior. These observations were consistent with earlier analyses. Furthermore, the recommended gradation had a narrower distribution than the standard gradation, enabling tighter control over the aggregate proportions during mixture design.

**Table 23:** Recommended gradation range.

Gradation	Pass rate (%)									
	19mm	13.2mm	9.5mm	4.75mm	2.36mm	1.18mm	0.6mm	0.3mm	0.15mm	0.075mm
Recommended	100	65 ~ 72	43 ~ 51	26 ~ 33	20.5 ~ 26.5	15 ~ 19	10.5 ~ 15	8.5 ~ 13	6.8 ~ 10.8	6 ~ 10
Standard	100	70 ~ 92	60 ~ 80	34 ~ 62	20 ~ 48	13 ~ 36	9 ~ 26	7 ~ 18	5 ~ 14	4 ~ 8

To evaluate the performance of the mixtures with both the standard and recommended gradations, four tests were conducted: dynamic stability, SCB low-temperature fracture toughness, four-point bending fatigue life, and immersion Marshall stability. The results are summarized in Table 24. Compared with the standard gradation, the asphalt mixture prepared with the recommended gradation exhibited improvements of 35% in dynamic stability, 28% in fracture toughness, 21% in fatigue life, and 4% in immersion Marshall stability. These results demonstrated the superior performance and application potential of the recommended gradation.

**Table 24:** Pavement performance of asphalt mixture.

Performance indicators		Recommended gradation	Standard gradation	Performance ratio
Dynamic stability	*A*_*V*_ (times)	3310	2451	1.35
	*C*_*V*_ (%)	4.72	2.16	6.85
Fracture toughness	*A*_*V*_ (MP·m^0.5^)	1.44	0.89	1.28
	*C*_*V*_ (%)	3.34	8.12	5.93
Fatigue life	*A*_*V*_ (cycles)	20661	17076	1.21
	*C*_*V*_ (%)	7.68	4.01	2.89
Immersion Marshall stability	*A*_*V*_ (%)	93.5	89.9	1.04
	*C*_*V*_ (%)	6.43	5.27	4.66

### Environmental impact comparison analysis

Using life cycle assessment (LCA) [41], the study quantified the carbon emissions associated with the raw material production stage of the optimized RAP-containing asphalt mastic (mineral filler–binder ratio = 1.4, FRAP–fine aggregate ratio = 50:50, *K* value = 0.65). Two scenarios were considered to compare the carbon emissions associated with the raw material production stage: (a) Scenario A was the baseline case, corresponding to conventional asphalt mastic without RAP; (b) Scenario B was an asphalt mastic containing 50% RAP with the optimized composition proposed in this paper. This scenario reflected not only the RAP substitution effect but also the reduction in the virgin asphalt binder demand resulting from mastic structure optimization. It was assumed that the energy consumption and emissions associated with the transportation, construction, and use phase were the same for the two scenarios to isolate and highlight the environmental differences arising solely from material production and composition. The material compositions of each scenario per ton of asphalt mastic are summarized in Table 25.

Carbon emission factors are expressed in terms of CO_2_ equivalent (CO_2_e) to represent the global warming potential of multiple greenhouse gases combined. The carbon emission factors of the key raw materials used in the calculations are listed in Table 25. The emission factor for RAP only accounted for the energy consumption associated with crushing and sieving.

**Table 25:** Material composition of asphalt mastic per ton.

Material	Scenario A (kg)	Scenario B (kg)
Virgin asphalt binder	120	100
Mineral filler	88	140
Fine aggregate	792	380
RAP	0	380

Based on the material compositions listed in Table 25 and the carbon emission factors listed in Table 26, the total carbon emissions during the raw material production stage were calculated and summarized for the two scenarios, as presented in Table 27.

**Table 26:** Carbon emission factors of materials.

Material	Emission factor(kg CO_2_e/t)	Source
Virgin asphalt binder	373	Eurobitume [42]
Mineral filler	7.36	Zhang et al. [43]
Fine aggregate	2.06	Gruber et al. [44]
RAP	0.36	Gruber et al. [44]

**Table 27:** Comparison of carbon emissions during the raw material production stage.

Scenario	Carbon emissions(kg·CO_2_e/t mastic)
Scenario A	47.04
Scenario B	39.25

As shown in Table 27, compared with conventional asphalt mastic without RAP (Scenario A), an asphalt mastic containing 50% RAP with the optimized composition (Scenario B) significantly reduced carbon emissions during the raw material production stage. Specifically, the total carbon emissions decreased from 47.04 kg CO_2_e/t in Scenario A to 39.25 kg CO_2_e/t in Scenario B, corresponding to a reduction of approximately 16.5%. These findings indicated that optimizing the composition of RAP-containing asphalt mastic offers an effective approach to mitigating the environmental impact associated with the material production phase of asphalt pavements.

## Conclusions

In this study, the compositional design of asphalt mastic containing fine recycled asphalt pavement materials (FRAP) and its influence on mixture performance were systematically investigated. The main conclusions are as follows:

- An optimal asphalt mastic composition was identified when the mineral filler–binder ratio was 1.4, the FRAP–fine aggregate ratio was 50:50 by mass, and the *K* value was 0.65, resulting in improved dynamic stability and splitting strength.
- Statistical analysis indicated that the mineral filler–binder ratio was the dominant factor governing asphalt mastic performance, followed by the FRAP–fine aggregate ratio. The influence of the *K* value was comparatively less pronounced, highlighting the importance of mineral binder–filler interactions in FRAP-containing mastic design.
- At the mixture-structure level, a coarse aggregate-to-asphalt mastic ratio of 75:25 provided the most balanced overall performance, reflecting effective coordination between the aggregate skeleton and the mastic phase.
- Asphalt mixtures designed using the proposed mastic composition and structural ratio exhibited significantly enhanced resistance to rutting and cracking compared with the standard gradation, demonstrating clear advantages for engineering applications with high recycled asphalt pavement content.

In future studies, the rheological aging characterization of asphalt binder, along with microstructural analysis and multi-temperature evaluation of RAP asphalt mastic, will be conducted to enhance the mechanistic understanding and to assess the long-term durability of the proposed material design.

## Supporting information

S1 File Data. This file includes all the test data of the asphalt materials.
(DOCX)

S2 File Graphic abstract. (DOCX)
