# Supplementary material for: Composition optimization of mastic in recycled asphalt mixtures based on pavement performance
Source: PLoS One. 2026 Mar 6;21(3):e0344180. doi: 10.1371/journal.pone.0344180 (PMC12965581; doi:10.1371/journal.pone.0344180)
Supplement: S1 File — This file includes all the test data of the asphalt materials. (DOCX) [file pone.0344180.s001.docx]

**Performance of asphalt mastic under different mineral filler–binder**

| Mineral filler–binder ratio | DSR | | BBR | |
| --- | --- | --- | --- | --- |
|  | *A_V_* (kPa) | *C_V_* (%) | *A_V_* (MPa) | *C_V_* (%) |
| 0.6 | 2.51 | 3.74 | 125 | 6.21 |
| 0.8 | 2.60 | 8.41 | 140 | 5.74 |
| 1.0 | 3.00 | 5.63 | 160 | 7.80 |
| 1.2 | 3.50 | 2.56 | 280 | 4.74 |
| 1.4 | 3.90 | 7.25 | 420 | 3.42 |
| 1.6 | 4.10 | 5.12 | 465 | 4.83 |
| 1.8 | 4.25 | 6.48 | 500 | 2.65 |
| 2.0 | 4.32 | 5.69 | 505 | 5.16 |

**Test results of splitting strength at different *K* values**

| *K* value | Splitting strength | |
| --- | --- | --- |
|  | *A_V_* (MPa) | *C_V_* (%) |
| 0.50 | 0.65 | 3.18 |
| 0.55 | 0.68 | 2.52 |
| 0.60 | 0.75 | 2.27 |
| 0.65 | 0.85 | 4.71 |
| 0.70 | 0.82 | 3.58 |
| 0.75 | 0.80 | 4.49 |
| 0.80 | 0.76 | 6.83 |
| 0.85 | 0.72 | 4.22 |

**Test results of asphalt mastic with different FRAP**–**fine aggregate ratios**

| FRAP–fine aggregate ratio | Dynamic stability | | Splitting strength | |
| --- | --- | --- | --- | --- |
|  | *A_V_* (times) | *C_V_* (%) | *A_V_* (MPa) | *C_V_* (%) |
| 80:20 | 690 | 6.25 | 0.57 | 5.83 |
| 70:30 | 720 | 5.13 | 0.60 | 3.67 |
| 60:40 | 880 | 7.32 | 0.68 | 1.54 |
| 50:50 | 1480 | 6.55 | 0.92 | 2.89 |
| 40:60 | 1020 | 4.90 | 0.70 | 2.36 |
| 30:70 | 600 | 8.62 | 0.45 | 5.93 |
| 20:80 | 580 | 3.47 | 0.41 | 4.35 |

**Orthogonal test results**

| Test Number | Dynamic stability | | Splitting strength | |
| --- | --- | --- | --- | --- |
|  | *A_V_* (times) | *C_V_* (%) | *A_V_* (MPa) | *C_V_* (%) |
| 1 | 873 | 3.62 | 0.97 | 6.28 |
| 2 | 1226 | 7.27 | 1.17 | 9.12 |
| 3 | 1376 | 2.15 | 1.30 | 2.57 |
| 4 | 859 | 5.83 | 0.81 | 5.48 |
| 5 | 791 | 1.44 | 0.91 | 7.93 |
| 6 | 1489 | 8.90 | 1.45 | 3.27 |
| 7 | 1159 | 4.36 | 1.12 | 1.99 |
| 8 | 960 | 6.71 | 0.85 | 8.53 |
| 9 | 1139 | 9.38 | 0.94 | 4.18 |
| 10 | 1109 | 2.81 | 1.08 | 6.91 |
| 11 | 934 | 5.17 | 0.96 | 6.08 |
| 12 | 1267 | 7.53 | 1.16 | 2.33 |
| 13 | 1321 | 3.98 | 1.05 | 5.60 |
| 14 | 1042 | 1.74 | 1.01 | 7.37 |
| 15 | 986 | 8.33 | 0.90 | 7.02 |
| 16 | 1068 | 4.76 | 0.95 | 3.37 |

**Verification test results of the optimal combination**

| Test Number | Dynamic stability | | Splitting strength | |
| --- | --- | --- | --- | --- |
|  | *A_V_* (times) | *C_V_* (%) | *A_V_* (MPa) | *C_V_* (%) |
| Ⅰ | 1464 | 3.21 | 1.42 | 4.33 |
| Ⅱ | 1279 | 9.67 | 1.25 | 1.19 |
| Ⅲ | 1252 | 7.93 | 1.23 | 8.55 |
| Ⅳ | 1405 | 5.48 | 1.37 | 6.74 |
| Ⅴ | 1386 | 1.82 | 1.35 | 2.35 |
| Ⅵ | 1453 | 2.07 | 1.39 | 7.12 |
| Ⅶ | 1423 | 6.54 | 1.41 | 5.48 |

**Performance of asphalt mixtures at different coarse aggregate-to-asphalt mastic ratios**

| Coarse aggregate-to-asphalt mastic | Dynamic stability | | Fracture toughness | | Fatigue life | | Immersion Marshall stability | |
| --- | --- | --- | --- | --- | --- | --- | --- | --- |
|  | *A_V_* (times) | *C_V_* (%) | *A_V_* (MP·m^0.5^) | *C_V_* (%) | *A_V_* (cycles) | *C_V_* (%) | *A_V_* (%) | *C_V_* (%) |
| 90:10 | 1922 | 2.47 | 0.99 | 3.39 | 79864 | 5.91 | 75.8 | 6.28 |
| 85:15 | 2738 | 3.08 | 1.20 | 1.92 | 123574 | 6.72 | 86.1 | 5.19 |
| 80:20 | 3131 | 5.93 | 1.32 | 4.74 | 195006 | 7.03 | 91.5 | 4.84 |
| 75:25 | 3410 | 1.64 | 1.48 | 1.31 | 210250 | 5.47 | 93.1 | 6.29 |
| 70:30 | 2770 | 4.56 | 1.14 | 2.36 | 177318 | 6.28 | 91.2 | 7.89 |
| 65:35 | 2110 | 2.18 | 0.92 | 4.02 | 142424 | 5.66 | 88.9 | 1.58 |
| 60:40 | 1653 | 3.95 | 0.83 | 3.44 | 111210 | 6.95 | 87.1 | 8.09 |

**Pavement performance of asphalt mixture**

| Performance indicators | | Recommended gradation | Standard gradation | Performance ratio |
| --- | --- | --- | --- | --- |
| Dynamic stability | *A_V_* (times) | 3310 | 2451 | 1.35 |
|  | *C_V_* (%) | 4.72 | 2.16 | 6.85 |
| Fracture toughness | *A_V_* (MP·m^0.5^) | 1.44 | 0.89 | 1.28 |
|  | *C_V_* (%) | 3.34 | 8.12 | 5.93 |
| Fatigue life | *A_V_* (cycles) | 20661 | 17076 | 1.21 |
|  | *C_V_* (%) | 7.68 | 4.01 | 2.89 |
| Immersion Marshall stability | *A_V_* (%) | 93.5 | 89.9 | 1.04 |
|  | *C_V_* (%) | 6.43 | 5.27 | 4.66 |
