# Supplementary figures and images for: Composition optimization of mastic in recycled asphalt mixtures based on pavement performance
Source: PLoS One. 2026 Mar 6;21(3):e0344180. doi: 10.1371/journal.pone.0344180 (PMC12965581; doi:10.1371/journal.pone.0344180)

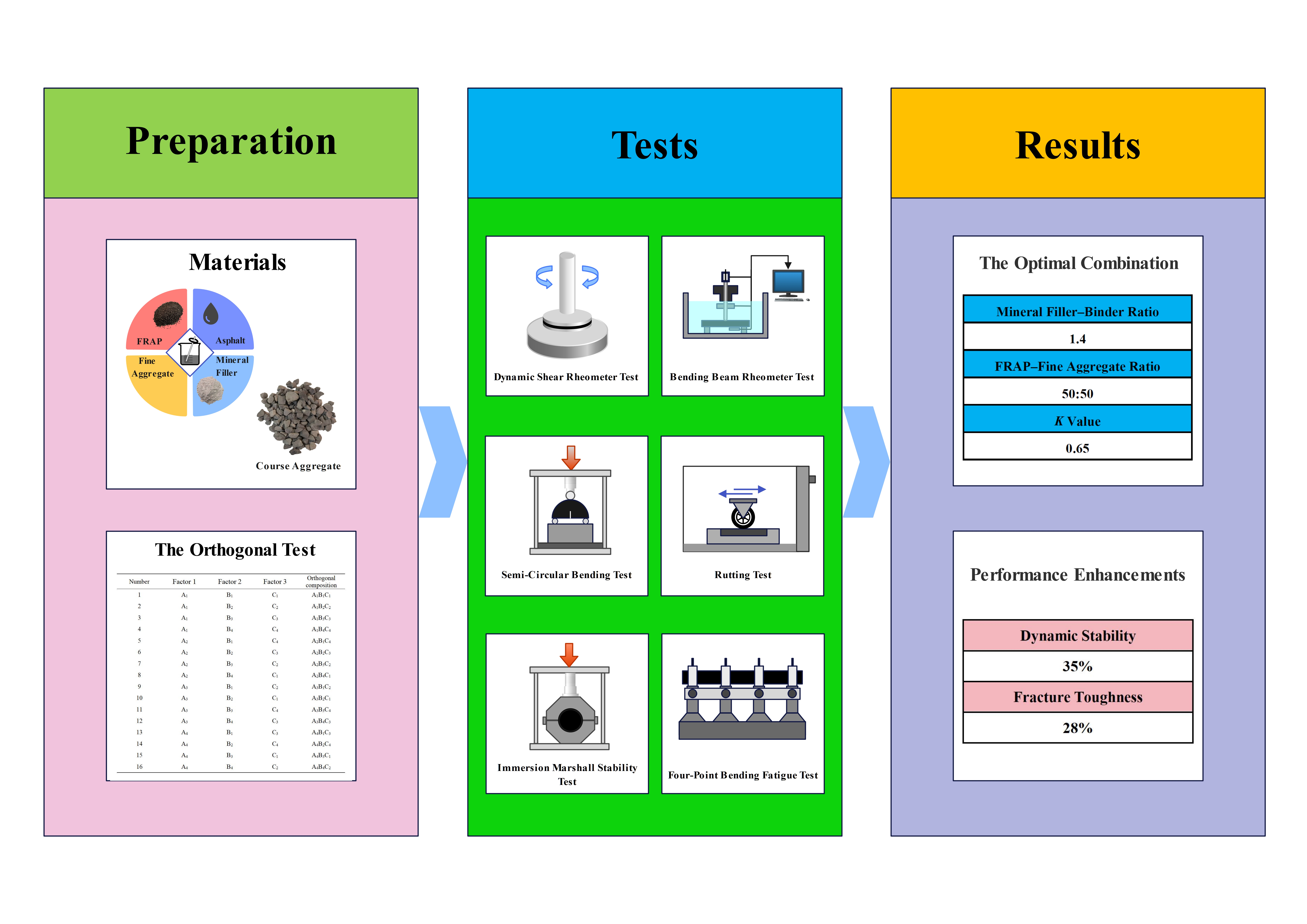


**Graphic abstract**

Supplement: S2 File — (DOCX) [file pone.0344180.s002.docx]
